# Cloning and characterization of the *CarbcL* gene related to chlorophyll in pepper (*Capsicum annuum* L.) under fruit shade stress

**DOI:** 10.3389/fpls.2015.00850

**Published:** 2015-10-13

**Authors:** Shu-Bin Wang, Shi-Lin Tian, Syed N. M. Shah, Bao-Gui Pan, Wei-Ping Diao, Zhen-Hui Gong

**Affiliations:** ^1^Institute of Vegetable Crops, Jiangsu Academy of Agricultural SciencesNanjing, China; ^2^College of Horticulture, Northwest A&F UniversityYangling, China; ^3^Department of Bioengineering, Huanghuai UniversityZhumadian, China; ^4^Department of Horticulture, Faculty of Agriculture, Gomal UniversityDera Ismail Khan, Pakistan

**Keywords:** capsanthin, *Capsicum annuum* L., fruit chlorophyll breakdown, fruit shading

## Abstract

Light is an important environmental factor for fruit development and ripening in pepper plant. Fruit bagging is a significant agrotechnology practiced for the illumination regulation of fruits; some previous researches have shown that fruit bagging could improve the appearance and external quality of fruits and cause them to mature early. However, it would decrease the intrinsic qualities of fruits; especially, fruit bagging could decrease the content of capsanthin in peppers. On the basis of these details, fruit bagging was used as the method of fruit shade stress in this study to explore the characteristics and molecular mechanisms of pepper fruit's color change under shade stress. By using cDNA-AFLP under fruit shading, a fragment related to fruit color was obtained. Next, the full-length coding sequence of the gene was cloned from the pepper fruits. Homologous gene alignment confirmed that the gene has high homology with the *rbcL* gene, named *CarbcL*. The function of the *CarbcL* gene was identified through virus-induced gene silencing (VIGS); it was found that the fruit color changed completely from green to red except for some residue of green fleck when *CarbcL* gene was silenced, and the green color of fruits had not fully faded in the control group and the empty vector group. The combine determination of chlorophyll content showed that *CarbcL* was involved in the metabolic control of chlorophyll in pepper fruits; subsequently, HPLC was used to determine the content of capsanthin in pepper fruit which the *CarbcL* gene was silencing, and it was also found that the content of capsanthin decreased appreciably. These results further confirmed that *CarbcL* gene was involved in the adjustment of chlorophyll and capsanthin.

## Introduction

In northwestern China, the light intensity is high in summer and the absorption of excess light by plants for photosynthesis may cause photo-oxidative damage. Light intensity can also affect the concentrations of carotenoids and ascorbic acid in fruits and vegetables (Asada, 1994). To overcome the this problem, farmers use shade netting in plastic greenhouses for cultivation, which not only decreases the quantity of light but also alters the quality of light to various extents and may affect other environmental factors (Asada, 1994). In recent years, the use of bagging and other methods has greatly improved the appearance and commercial value of fruits and has reduced the amount of pesticide residues on the products (Huang et al., 2009; Yang et al., 2009a). Fruit bagging is currently widely used on fruit trees to produce unblemished, high-quality fruits (Huang et al., 2009; Yang et al., 2009a; Xu et al., 2010). In the past few years, the large-scale use of bagging has become an important process in vegetable production (Seshachala and Tallapragada, 2013) and also has a significant effect on crop yield. For example, bagging in *Sorghum* was found to increase the carotenoid content by 8–184% and minimize the exposure of panicles to light (Blessin et al., 1963).

The above-mentioned researches focused only on the general quality and appearance of bagged fruits, but recent studies have shown that bagging also has certain negative effects on fruit development and may affect some qualitative traits (Huang et al., 2009; Xu et al., 2010). Huang et al. (2009) reported that bagging treatments in pear did not affect the total soluble sugar content but did decrease fruit organic acids. Their study further revealed that the levels of chlorophyll, carotenoids, flavonoids, and total phenols did not change much over time after bag removal; however, anthocyanin accumulated quickly (within 10 days) and remained at a constant level (Huang et al., 2009).

In our previous study, it was found that when pepper fruits were bagged at the fruit development stage (green stage), the green color faded earlier (earlier turned red) in bagged fruits than non-bagged fruits. This issue raised questions regarding, regulatory mechanism of fruit color at fruit development stages and genes involvement under shade stress. The Stay Green Rice (SGR) protein is a membrane-bound protein in the chloroplast and was first identified in rice. SGR protein is encoded by *SGR* gene and it affects chlorophyll degradation during natural and dark-induced leaf senescence in rice (Jiang et al., 2007; Aubry et al., 2008; Barry, 2009). The expression of *SGR* is closely associated with leaf senescence and fruit ripening (Barry et al., 2008; Yang et al., 2009b). Researches have showed that light affects expression of *ribulose-1,5-bisphosphate carboxylase/oxygenase* (*rbcL*) gene, light stimulates *rbcL* transcription, and increases transcription of *rbcL* (Kim et al., 2002; Sun et al., 2014). RuBisCo content is associated with mRNA level of *rbcL* (Suzuki et al., 2007). Previous researchers have focused on plant leaves, not fruits for the study of *rbcL* gene. There is also no report available on the link between chlorophyll and capsanthin. In the present study, we have cleared this phenomenon by using cDNA-AFLP. The pepper fruits were exposed to shade stress at green color stage, and gene expression and its molecular mechanism were studied.

## Materials and methods

### Ethics statement

This work did not involve endangered or protected species. We abided by the statement of ethical standards for submitted manuscripts, and the manuscript does not describe experiments involving human subjects or animals.

### Plant materials

Seeds of *Capsicum annuum* cv.R15 were provided by the *Capsicum* research group, Department of Horticulture, Northwest A&F University, P.R. China.

### Methods

#### Plant growth conditions

To break the dormancy of pepper seeds, pre-sowing treatment of pepper seeds was done as described previously (Tian et al., 2014a). When the seedlings had 8–10 true leaves, they were transplanted into plastic pots under artificial climate chamber; the environmental condition of the artificial climate chamber was day 25°C, 16 h/night 20°C, 8 h; the relative humidity was 35%, and the light intensity was 370,000 μmol.m^−2^.s^−1^. These plastic pots were arranged in a randomized complete block design in the artificial climate chamber. The specifications of plastic pot were 20 × 14 cm, the soil of pot comprised peat soil, sand, and perlite in the ratio 1:1:1, respectively, and Hoagland nutrient solution was used to provide nutrition for pepper plants.

#### Fruit shade stress

Pepper fruitlets were marked with labels at anthesis. Fruits of the same size and age were screened and were shaded (bagged) at the mature green stage (25 days after flowering). Specifications of bags: Single story, yellow, paper; length × width: 75 mm × 150 mm; Transmittance: 8.30%; Air permeability (mL/pa.s): 3.54. Control fruits of the same age and size were not shaded (unbagged) (Tian et al., 2013). After 7 days, fruits from each treatment (shaded or unshaded) were harvested; additional fruits were exposed to light for 4 h after shade removal and then harvested. The harvested fruits were immediately placed on ice for transport to the laboratory, where the seeds were removed and the peels cut into small pieces and stored at −80°C for further analysis (Tian et al., 2014a).

#### cDNA-AFLP analysis of transcript-derived fragments (TDFs) under fruit shade stress

Total RNA was extracted separately as described previously (Dal Cin et al., 2005) from each sample and used as a template with oligo (dT) 18 as a primer. First-strand cDNAs were synthesized by AMV reverse transcriptase, and the complementary strand was replicated by *E. coli* DNA polymerase I after the fragments were digested with restriction enzymes (*EcoR*I and *Mse*I) and ligated. Pre-amplification was performed with the following pre-amplification primers: *EcoR*I: 5′-GACTGCGTACCAATTCA-3′, *Mse*I: 5′-GATGAGTCCTGAGTAAC-3′. The resulting products were used as templates for selective amplification (Vuylsteke et al., 2007; Yu et al., 2011) with selective primers (Supplementary Table 1). The PCR products were resolved via electrophoresis on a 4% denaturing polyacrylamide gel, which was then silver-stained and scanned. The fragments of interest were excised from the gel and re-amplified using the same primer combinations and PCR conditions used in the selective amplification (Vuylsteke et al., 2007; Yu et al., 2011). All differentially expressed gene sequences were analyzed using the basic local alignment search tool (BLAST) at the National Center for Biotechnology Information (NCBI) website (http://www.ncbi.nlm.nih.gov/blast/) (Vuylsteke et al., 2007; Yu et al., 2011). Among these TDFs, the TDFs related to fruit color were selected for further experiment. The PCR products were subsequently cloned into a pMD19-T vector plasmid (TaKaRa Biotechnology (Dalian) Co., Ltd.) for sequencing.

#### Virus vector construction

**Figure 1:**
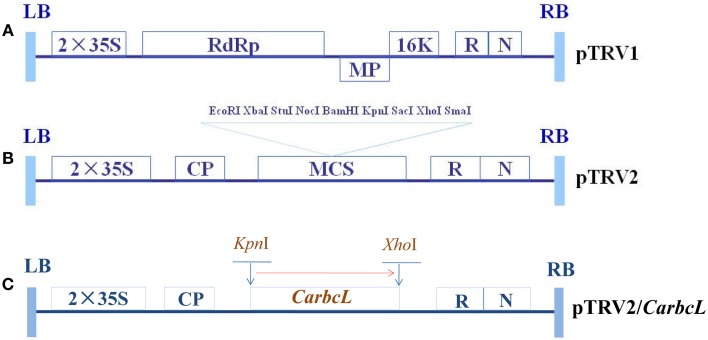
**Schematic representation of TRV and recombinant TRV vectors carrying the target genes**. **(A)** The structure of pTRV1; **(B)** The structure of pTRV2; **(C)** The structure of TRV-*CarbcL*. LB, left border of the T-DNA; RB, right border of the T-DNA; 2 × 35S: two copies of the cauliflower mosaic virus 35S promoter; CP, coat protein; RdRp, RNA-dependent RNA polymerase; MP, movement protein; 16K, 16 KDa protein; R, ribozyme; N, NOS terminator; and MCS, multiple cloning site.

Primers were designed based on the structure of TRV; the upstream primer carried a *Kpn*I site, whereas the downstream primer carried an *Xho*I site. These primers were used to transfer the target genes into the TRV vector (F: 5′-CGCGGTACC TCGGTCCATACAGTTGTC-3′; R: 5′-GCGCTCGAG GAGT TGTAGGGAGGGATT-3′). Restriction endonucleases (*Kpn*I and *Xho*I) were used to digest the pTRV2 virus vector and the target gene at 37°C overnight (Wang et al., 2013; Tian et al., 2014a), and the digestion products were separated by electrophoresis and ligated to the pTRV2 viral vector using T4 DNA polymerase; the ligation products were transformed into *E. coli* DH5α cells, and colony PCR was used to detect the target gene fragments. A schematic representation of this vector is shown in Figure 1. The *CarbcL* gene was cloned into the pTRV2 vector using four fragments from the 3′ end of the open reading frame (Figure 1). The pTRV/00 (empty vector) was used as a negative control.

#### Cloning of target gene fragments

Gene fragments used for silencing gene expression must be 150–500 bp in length (Wang et al., 2013). Primers for the PCR amplification of *CarbcL* were designed according to the sequences of carotenoid-related genes (F: 5′-CGCGGTACC TCGGTCCATACAGTTGTC-3′; R: 5′-GCGCTCGAG GAGTTG TAGGGAGGGATT-3′). Total fruit RNA was purified with Trizol and utilized as a template for cDNA synthesis, which was then used as a template for PCR amplification. The PCR products were separated via agarose gel electrophoresis, and target gene fragments were recovered. The recovered products were then ligated into the cloning vector pMD19-T via overnight incubation with T4 DNA ligase at 16°C, and the ligation reactions were then transformed into *E. coli* DH5α cells. Plasmids (pTRV2 and PMD19-T) carrying the target genes were digested individually with *Kpn*I and *Xho*I, and the pTRV2 restriction fragments and target gene fragments were then ligated together (Figure 1).

#### Genetic transformation of agrobacterium

The pTRV1 and pTRV2 vectors were introduced into *Agrobacterium* strain GV3101 by freeze-thaw method (Wang et al., 2013). The presence of the vectors in the bacterium was confirmed by PCR, and the bacterial cultures were stored for future use in further experiments.

#### Virus-induced gene silencing (VIGS) and TRV vector inoculation into fruits

The fruits were washed with distilled water individually, dried at room temperature, and the stalks of the fruits were sealed with melted wax. Then the fruits were sterilized with 75% alcohol for 30 s and washed three times with sterilized distilled water in sterilized laminar flow hood. The pTRV1, pTRV2, or pTRV2/*CarbcL* vector was mixed with *Agrobacterium tumefaciens* strain GV3101 at a 1:1 ratio. Cultures of *Agrobacterium* containing pTRV1, pTRV2/00, and pTRV2/*CarbcL* (OD_600_ = 1.0) were injected into detached pepper fruits. The TRV vector culture (0.5 ml) was injected at the base of the stalk of each fruit with help of syringe without a needle (Tian et al., 2014a).

The fruits were placed on sterilized filter papers on a stainless steel plate and covered with food-grade cling-film wrap. The plate was placed in a dark chamber (18°C with 35% RH) for 2 days. After 2 days, the treated fruits were transferred to a growth chamber (23°C/20°C under a 16 h light/8 h dark cycle with 35% relative humidity). Control (TRV/00) and silenced (TRV/*CarbcL*) fruits were used for analyses at 15 days after inoculation (Tian et al., 2014a).

### Chlorophyll extraction

Chlorophyll content was extracted from 10 g fruit sample in 30 mL of acetone. The process was repeated till no color left. The extracts were poured into a separating funnel and treated with 100 mL of diethyl ether. Then, 100 mL of sodium chloride (100 g/L) were added to the mixture. The ethereal layer was separated from the acetone, washed three times with 100 mL of 50 g/L anhydrous Na_2_SO_4_, filtered through a bed of anhydrous Na_2_SO_4_, and left for dryness under vacuum in a rotary evaporator (Switzerland) at 30°C. The residue was dissolved in 5 mL of acetone and stored at −20°C.

#### Chlorophylls analysis for HPLC

HP1100 Hewlett-Packard liquid chromatograph fitted with an automatic injector and a diode array detector was used for quantification of chlorophylls. A stainless steel column (25 × 0.46 cm), packed with 5 μm of C18 HPLC column. The solution of pigments in acetone was centrifuged at 12,000 g (Eppendorf China LTD, Germany) prior to injection into the chromatograph (20 μL) (Roca and Miänguez-Mosquera, 2006).

Quantification of the chlorophyll pigments was carried out as described previously (Roca and Miänguez-Mosquera, 2006). Separation was performed using an elution gradient (flow rate) 2 mL min^−1^ with the mobile phases (A) water/ion pair reagent/methanol (1:1:8 v/v/v) and (B) acetone/methanol (1:1 v/v). The ion pair reagent was 0.05 mol/L tetrabutylammonium acetate and 1 mol/L ammonium acetate in water. Detection was performed simultaneously at 666 nm for series *a* and 650 nm for series *b*. Response factors were calculated for each individual pigment by performing calibration plots (peak area ratio vs. concentration ratio) in the presence of a known amount of the pure standard solutions. Chlorophylls (chl) *a* and *b* were purchased from Sigma. Chlorophyllide was formed by enzymatic de-esterification of chl. The reaction mixture contained 100 mmol/L Tris-HCl (pH 8.5) containing 0.24% (w/v) Triton X-100, chl *a* dissolved in acetone, and crude enzymatic extract from fruits in a 5:1:5 (v/v/v) ratio. The C-13 epimer of Chl (*a* or *b*) was prepared by treatment with chloroform. 13^2^-OH-Chl (*a* or *b*) was obtained by selenium dioxide oxidation of Chl at reflux heating for 4 h in pyridine solution under argon. All Mg-free derivatives were obtained from the corresponding Chl parent dissolved in diethyl ether by acidification with 2–3 drops of 5 mol/L HCl. All standards were purified by normal phase (NP) and reversed phase (RP) thin-layer chromatography (TLC) (Roca and Miänguez-Mosquera, 2006).

#### Capsanthin extraction and analysis

Capsanthin was extracted as described previously (Tian et al., 2014a). A 5.0 g sample of pericarp tissue was extracted with 5 ml of acetone containing 0.1% butylated hydroxytoluene (BHT). After shaking and incubation on ice in the dark for 10 min, the mixture was centrifuged at 3500 rpm for 10 min at room temperature and the extract was transferred to a clean tube. Samples were re-extracted twice with 5.0 ml of acetone containing 0.1% BHT. Pooled extracts were dried under a nitrogen flow, and the tubes were sealed and stored at −20°C until high pressure liquid chromatography (HPLC) analysis. HPLC was performed as described previously (Tian et al., 2014a). For HPLC, samples (20 μL) were analyzed on a shim-pack VP-ODS C-18 HPLC column (5 μm, 150 × 4.6 mm). The eluent consisted of acetonitrile: 2-propanol: water in the ratio of 39:53:8 (A), respectively, and acetonitrile: 2-propanol in the ratio of 60:40 (B), respectively. The gradient profile was set 0–30 min from 0 to 100% B. The flow rate was set at 0.3 mL/min and the column temperature at 40°C. Standard solution of capsanthin (0.001–0.1 mg/mL) was used to make calibration curve at 454 nm. The capsanthin was identified by their absorption spectra as captured by the photodiode array detector and HPLC retention times in comparison with authentic standards. In addition, samples were spiked with standards to verify the identity of sample peaks with similar retention times. Capsanthin was purchased from Extrasynthèse (Genay, France), and it was used as authentic standards. The standard was handled under low light conditions on ice. Standard solution of capsanthin standard consisted methanol and acetonitrile in the ratio 1:1 (V/V). Aliquots were diluted in methanol:acetonitrile (1:1) solution to provide standard concentrations (Tian et al., 2014a). Each value was the mean of three biological replicates.

#### RNA isolation and qRT-PCR analysis

The fruits (treated and non-treated) were used for total RNA extraction. RNA was extracted through Trizol (Invitrogen) and quantified through NanoDrop spectrophotometer (Nano Drop 2000c, Thermo Scientific, Wilmington, USA) (Wang et al., 2009, 2013; Tian et al., 2014b). First-strand cDNA was synthesized from 500 ng of total RNA using a PrimeScript™ Kit (TaKaRa, Bio Inc., China) following the manufacturer's protocols. qRT-PCR was performed using SYBR® Premix Ex Taq™ II (TaKaRa, Bio Inc., China) with 10.0 μl SYBR® Premix Ex Taq™ II, 2.0 μl diluted cDNA, and 0.8 μl forward and reverse primers with total volume of 20 μl (Wang et al., 2013; Tian et al., 2014a,b). The qRT-PCR condition was maintained at 95°C for 1 min, followed by 45 cycles at 95°C for 10 s, 50°C for 30 s, and 72°C for 20 s, and primers F:5′-GAGTTGTAGGGAGGGATT-3′; R:5′-TCGGTCCATACAGTTGTC-3′, while *Ubi*3 (AY486137.1) was used as an internal control (Wan et al., 2011). The ΔΔCt method was used for relative expression levels of each gene (Livak and Schmittgen, 2001). The treatments were replicated thrice as well as experiment to minimize the error.

### Statistical analysis

SAS 6.12 software (SAS Institute, Gary, North Carolina) was used for data analysis. All measured values were presented as mean ± standard error of the means. Duncan's multiple-range test was chosen, and LSR (Least significant ranges) analysis at 5% significantly different is shown in lowercase letters (a and b) (Duncan, 1995). Means with the same letter are not significantly different. Figures were drawn using Sigma Plot 10.0 software; all experiments were carried out in triplicate.

## Results

### Identification of differentially expressed TDFs

**Figure 2:**
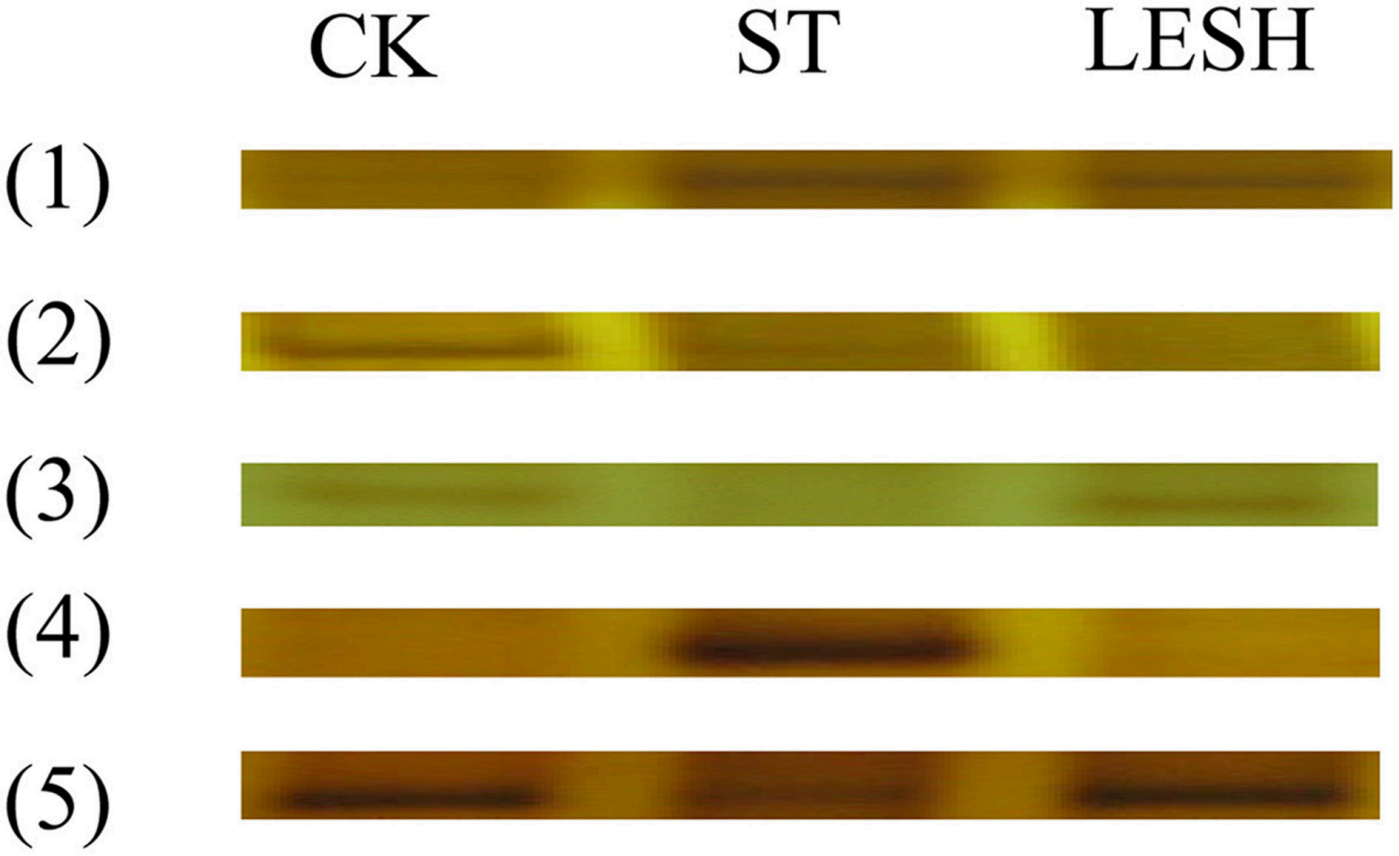
**The results of selective amplification**. CK, Control; ST, shade treatment; LESH, light exposure after shade removal. The differentially expressed bands were divided into five groups: (1) expressed during shade treatment (ST) and during light exposure after shade removal (LESH) but not in the control (CK); (2) expressed in ST and CK but not LESH; (3) expressed in CK and LESH but not ST; (4) expressed in ST but not CK and LESH; and (5) expressed in ST, LESH and CK at different levels.

Primers (64 pairs) were randomly applied for selective cDNA-AFLP amplification (Supplementary Table 1). The 80 clear bands with differential expression were revealed by analyzing the shaded fruits, the fruits that were exposed to light for 4 h after shade removal, and the control fruits (unbagged). These bands were divided into five groups: (1) expressed during shade treatment (ST) and during light exposure after shade removal (LESH) but not in the control (CK); (2) expressed in ST and CK but not LESH; (3) expressed in CK and LESH but not ST; (4) expressed in ST but not CK and LESH; and (5) expressed in ST, LESH, and CK at different levels. All of differential expression bands were excised from SDS-polyacrylamide gel and to renew amplifies by PCR using these differential bands as templates; finally, these differential bands were separated by agarose gel electrophoresis, and the new bands were excised from agarose gel and transformed into *Escherichia coli* for DNA sequencing. Figure 2 shows the differentially expressed bands.

### Sequence analysis of TDFs

A total of 80 reproducible TDFs were identified with 64 primer combinations tested. All of these TDFs were cloned and sequenced as described previously (Vuylsteke et al., 2007; Yu et al., 2011; Lin et al., 2014). All differentially expressed gene sequences were analyzed using the basic local alignment search tool (BLAST) at the National Center for Biotechnology Information (NCBI) website (http://www.ncbi.nlm.nih.gov/blast/). Among these TDFs, 27 bands were differently expressed, and the homology results were obtained with BLAST analysis (Supplementary Table 2). They were DNA-binding protein, ubiquitin-proteasome, HTC in leaf, 26 s ribosomal RNA and transcription elongation factor, etc. (Supplementary Table 2), but only one TDF (GenBank: KT779503) was related to chlorophyll metabolism in fruit; the TDF is, perhaps, involved in the regulation and control of chlorophyll under fruit shade stress during pepper fruit development and ripening.

### Cloning and sequencing of the full-length gene

Based on BLAST analysis, the TDF (namely, subsequent *CarbcL* gene) related to chlorophyll metabolism in fruit was cloned. BLAST was used to identify a particular TDF relevant to chlorophyll synthesis. Based on the data obtained from GenBank and the pepper genome database (http://peppersequence.genomics.cn/page/species/blast) and RACE methods, we obtained a fragment of approximately 1364 bp. Sequencing and homology analyses showed that the full-length sequence is 1364 bp (GenBank: KT779528).

### Alignment of homologous genes

**Figure 3:**
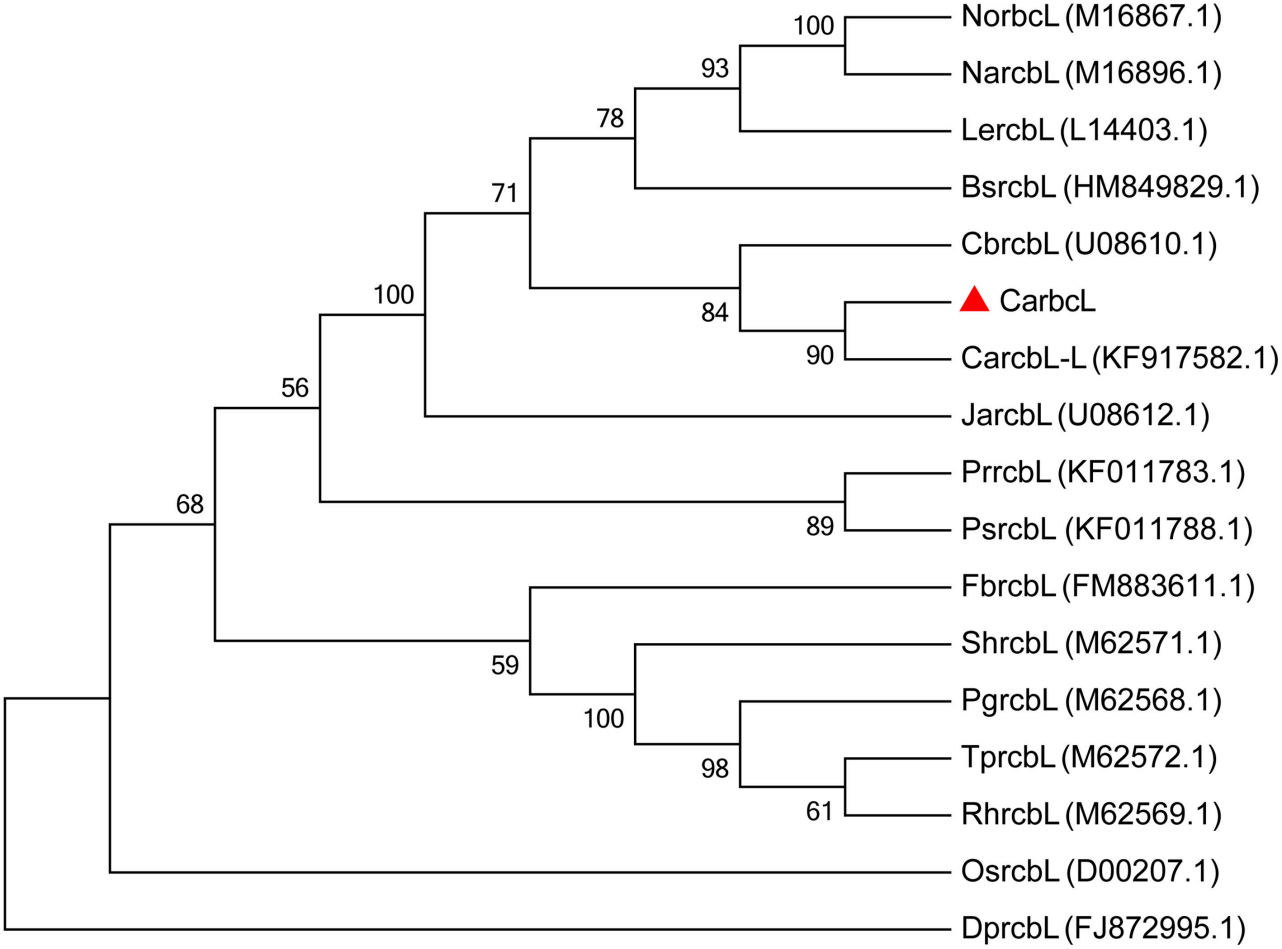
**The phylogenetic tree of ***CarbcL*** and other related sequences**. Numbers below the branches are the neighbor-joining bootstrap values; *CarbcL* gene was marked with “▴” symbol; GenBank accession numbers: (Tobacco) NorbcL, M16867.1; (*Lycopersicon esculentum*) LercbL, L14403.1; (*Brugmansia suaveolens*) BsrcbL, HM849829.1; (*Juanulloa aurantiaca*) JarcbL, U08612.1; (*Capsicum baccatum*) CbrcbL, U08610.1; (*Capsicum annuum*) CarcbL-L, KF917582.1; (*N. acuminata*) NarcbL, M16896.1; (*T. portulacastrum*) TprcbL, M62572.1; (*Durvillaea potatorum*) DprcbL, FJ872995.1; (*Pedicularis rex*) PrrcbL, KF011783.1; (*Pedicularis superba*) PsrcbL, KF011788.1; (*Fallopiax bohemica*) FbrcbL, FM883611.1; (*Portulaca grandiflora*) PgrcbL, M62568.1; (*S. halimifolium*) ShrcbL, M62571.1; (*R. humilis*) RhrcbL, M62569.1; (*Oryza sativa*) OsrcbL, D00207.1.

The base sequence (GenBank: KT779528) was put into NCBI/BLAST/blastn suite; it was found that the sequence of the cloned *Capsicum* gene is highly homologous to the ribulose-1,5-bisphosphate carboxylase/oxygenase gene (GenBank: KT779528) (Figure 3), with a similarity of 99%, so it was named *CarbcL*. Then, the gene sequence was evaluated using the pepper genome database (http://peppersequence.genomics.cn/page/species/blast). The sequence of *CarbcL* gene showed a 98% homology with the Capana12g001789 (http://peppersequence.genomics.cn).

### Functional analysis of *CarbcL* by VIGS

**Figure 4:**
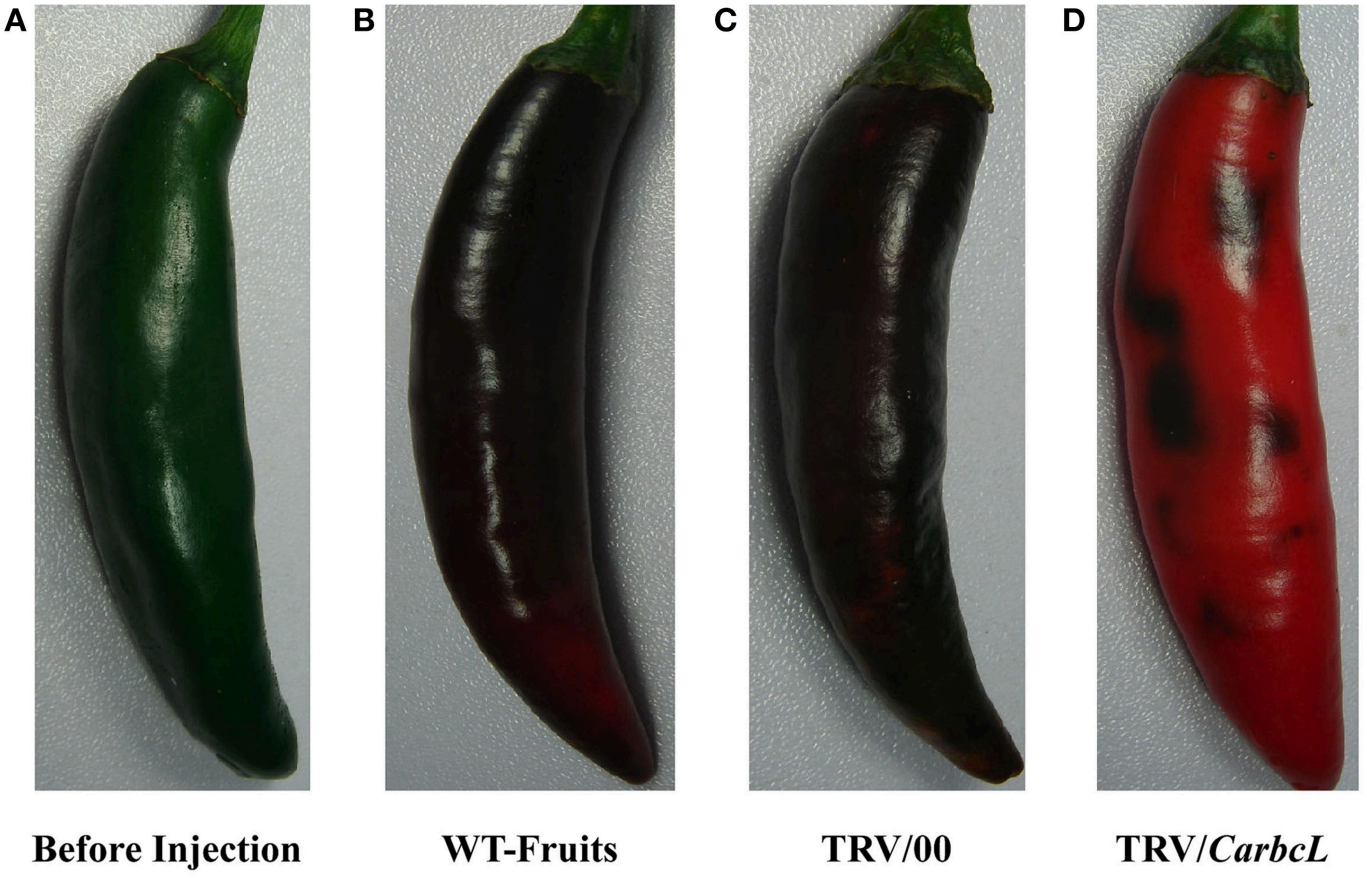
**Phenotypes of plants after ***CarbcL*** gene silencing**. **(A)** Before injection, the color of fruits before the sample was treated; **(B)** WT-Fruits, the color of fruits in the control group after 10 days; **(C)** TRV/00, the color of fruits in the empty vector group after 10 days; **(D)** TRV/*CarbcL*, the color of fruits in the treatment group (fruits were injected TRV/*CarbcL*) after 10 days of treatment.

**Figure 5:**
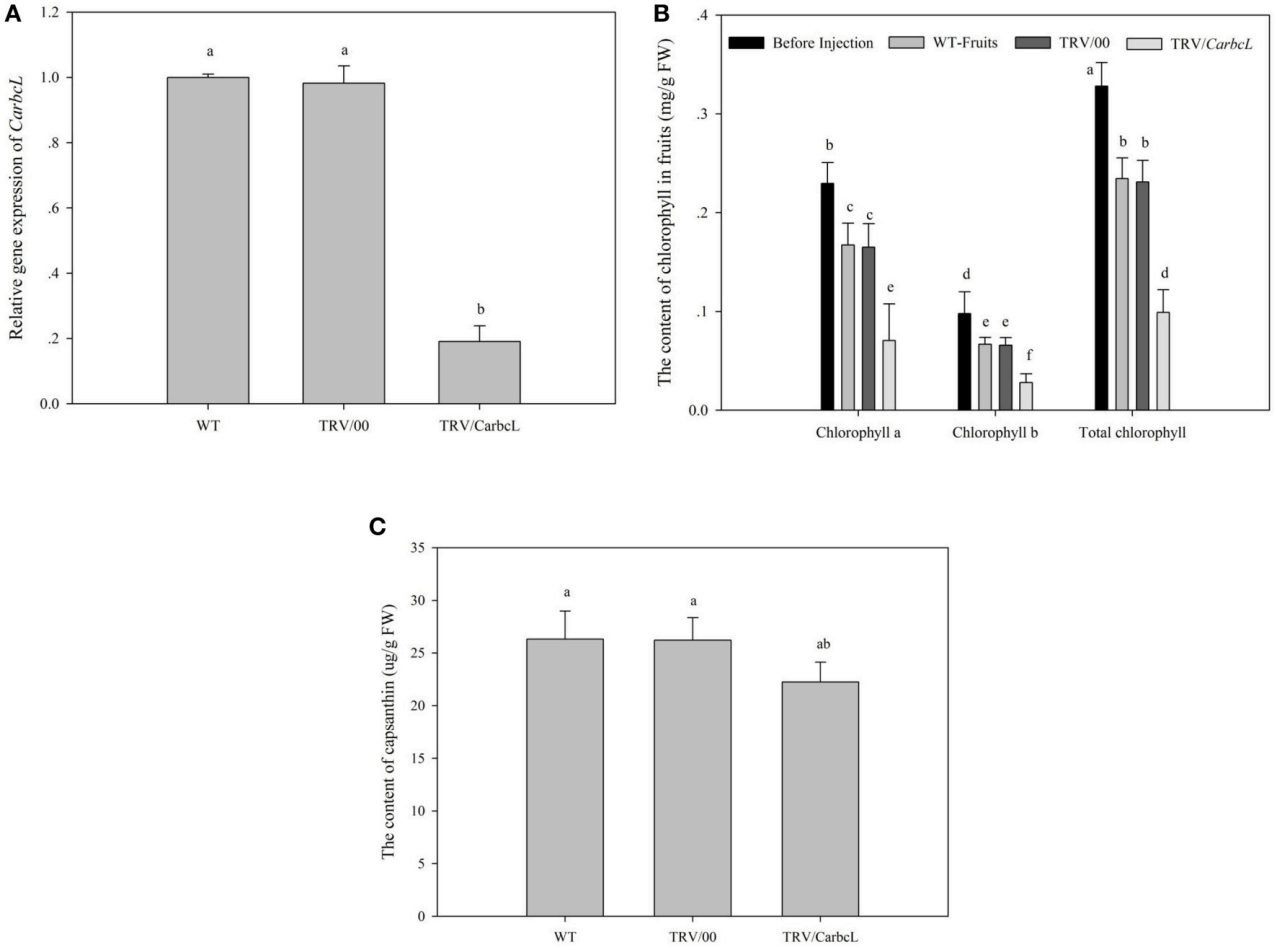
**Changes in gene expression level, chlorophyll content and fruit capsanthin content after gene silencing**. WT, the control group; TRV/00, the empty vector group; TRV/*CarbcL*, the treatment group. **(A)** The changes in gene expression level after silencing; *Ubi3* (AY486137.1) was used as an internal control by using qRT-PCR; **(B)** the changes in fruit chlorophyll content after gene silencing; Before injection, the content of chlorophyll before the sample was treated; WT-Fruits, the content of chlorophyll in the control group; TRV/00, the content of chlorophyll in the empty vector group after 10 days of treatment; TRV/*CarbcL*, the content of chlorophyll in the treatment group (fruits were injected TRV/*CarbcL*) after 10 days of treatment; **(C)** the changes in fruit capsanthin content after gene silencing; the content of capsanthin in the control group; TRV/00, the content of capsanthin in the empty vector group; TRV/*CarbcL*, the content of capsanthin in the treatment group; LSR analysis at 5% is shown in lowercase letters (a and b). Means with the same letter are not significantly different. All experiments were carried out in triplicate.

VIGS technology was used to further verify whether the gene is involved in the metabolic control of chlorophyll. TRV viral vectors, pTRV1 and pTRV2, were constructed as gene-silencing vectors for a preliminary determination of gene function in detached pepper fruits (Tian et al., 2014a). The detached pepper fruits in which the target gene was silenced showed symptoms of chlorosis on day 7 of treatment, and the symptoms were obvious on day 10. The fruit color was dark green before injection but changed after 10 days of treatment (Figure 4). The fruits in the control (WT fruits) and empty vector (TRV/00) groups were eventually turned brown, whereas the fruits in the silenced (TRV/*CarbcL*) group had turned red after 10 days of treatment, with only some green spots remaining. This result shows that the *CarbcL* gene was involved in the color control of fruit in pepper. RNA was extracted from the WT (control), TRV/00 (empty vector), and TRV/*CarbcL* (gene-silenced) fruits and reverse transcribed into cDNA using qRT-PCR to analyze the mRNA expression levels of *CarbcL*. As there were no differences in the levels of *CarbcL* mRNA between the WT fruits (control group) and TRV/00 (empty vector) fruits, the impact of the TRV virus vector was negligible. However, the expression level of *CarbcL* significantly decreased in the *CarbcL*-silenced fruits (Figure 5A), with a silencing efficiency of 81%. These results demonstrate that the green color of pepper fruits from the TRV/*CarbcL* plants faded due to silencing of the *CarbcL* gene.

### Expression analysis of *CarbcL* gene after VIGS

As everybody knows, the effect of gene silencing by VIGS was measured by the expression level of gene. After *CarbcL* gene was silenced, the expression level of *CarbcL* gene was analyzed in the WT fruits (control group) and the silenced (TRV/*CarbcL*) group. The result showed that the expression level of *CarbcL* gene significantly decreased after *CarbcL* gene was silenced; the expression level (TRV*/CarbcL*) was about 19% of the control group (WT) (Figure 5A). There were indications that the silencing effects of gene were ideal; it could be used as the basis of the gene function confirmation. From Figure 5A, it could reveal that there were no differences in the expression levels of *CarbcL* between the control group (WT) and the empty vector (TRV/00); the impact of the TRV virus vector was negligible. However, the expression level of *CarbcL* was very lower in the silenced (TRV/*CarbcL*) group than that of the control group (WT) (Figure 5A). This demonstrated that the fading of green color in pepper fruits was caused by *CarbcL* gene silencing (Figures 4D, 5A). This further proved that the green color of pepper fruit was closely related to *CarbcL* gene.

### Changes in the chlorophyll content of TRV/*CarbcL* pepper fruits

By comparing the chlorophyll content of the control fruit (WT-Fruits), the empty vector fruit (TRV/00), and fruit of gene silencing (TRV/*CarbcL*), it was found that the green color of fruit disappeared after 10 days except for some residue of green fleck when *CarbcL* gene was silenced; the color of fruit changed completely from green to red (Figures 4A,D); but the surface of the control group (WT-Fruits) kept a lot of green color, and the fruits presented a brownish red color (Figure 4B). To check again for the empty vector fruit (TRV/00), it was found that the color of the fruit was brownish red also (Figure 4C); There was no obvious difference in the color of fruits between the control fruit (WT-Fruits) and the empty vector fruit (TRV/00) (Figures 4B,C). This demonstrated that TRV vector had no impact on the color of fruit; the fading of green color of fruit (TRV/*CarbcL*) was due to *CarbcL* gene silencing (Figure 4D). For further study about the relationship between *CarbcL* and chlorophyll, the chlorophyll content was determined in the control (WT-Fruits) and the treatment (TRV/*CarbcL*). The result showed that the chlorophyll content was much lower in the *CarbcL*-silenced pepper fruits (TRV/*CarbcL*) than in the control fruits (WT-Fruits) (Figure 5B). These results showed that the *CarbcL* gene is involved in the metabolic control of chlorophyll in pepper fruits.

### Changes in the capsanthin content of pepper fruits when silencing *CarbcL*

After *CarbcL* gene silencing, the content of capsanthin was determined in the treatment group (TRV/*CarbcL*), the control group (WT), and the empty vector group (TRV/00). The result showed that the content of capsanthin was same basically in the control group (WT) and the empty vector group (TRV/00). A statistical analysis was made on capsanthin content among the three groups (WT, TRV/00, and TRV/*CarbcL*); it was found that the difference between the control group (WT) and the empty vector group (TRV/00) was not significant. However, when the content of capsanthin was determined in the treatment group (TRV/*CarbcL*), it was found that the content of capsanthin was lower than the control (WT) and the empty vector group (TRV/00). There were some differences in the content of capsanthin between the control group (WT) and treatment group (TRV/*CarbcL*) through statistical analysis. These results indicated that not only the normal metabolism of chlorophyll was affected but also the synthesis of capsanthin was also affected when *CarbcL* was silenced. The measuring result showed that the capsanthin content was reduced by 15.4% in the *CarbcL*-silenced pepper fruits than that in the control fruits (Figure 5C). This result also confirms that the *CarbcL* gene is also associated with synthesis of capsanthin in pepper fruits.

### Further confirmation of *CarbcL* gene expression under shade stress

**Figure 6:**
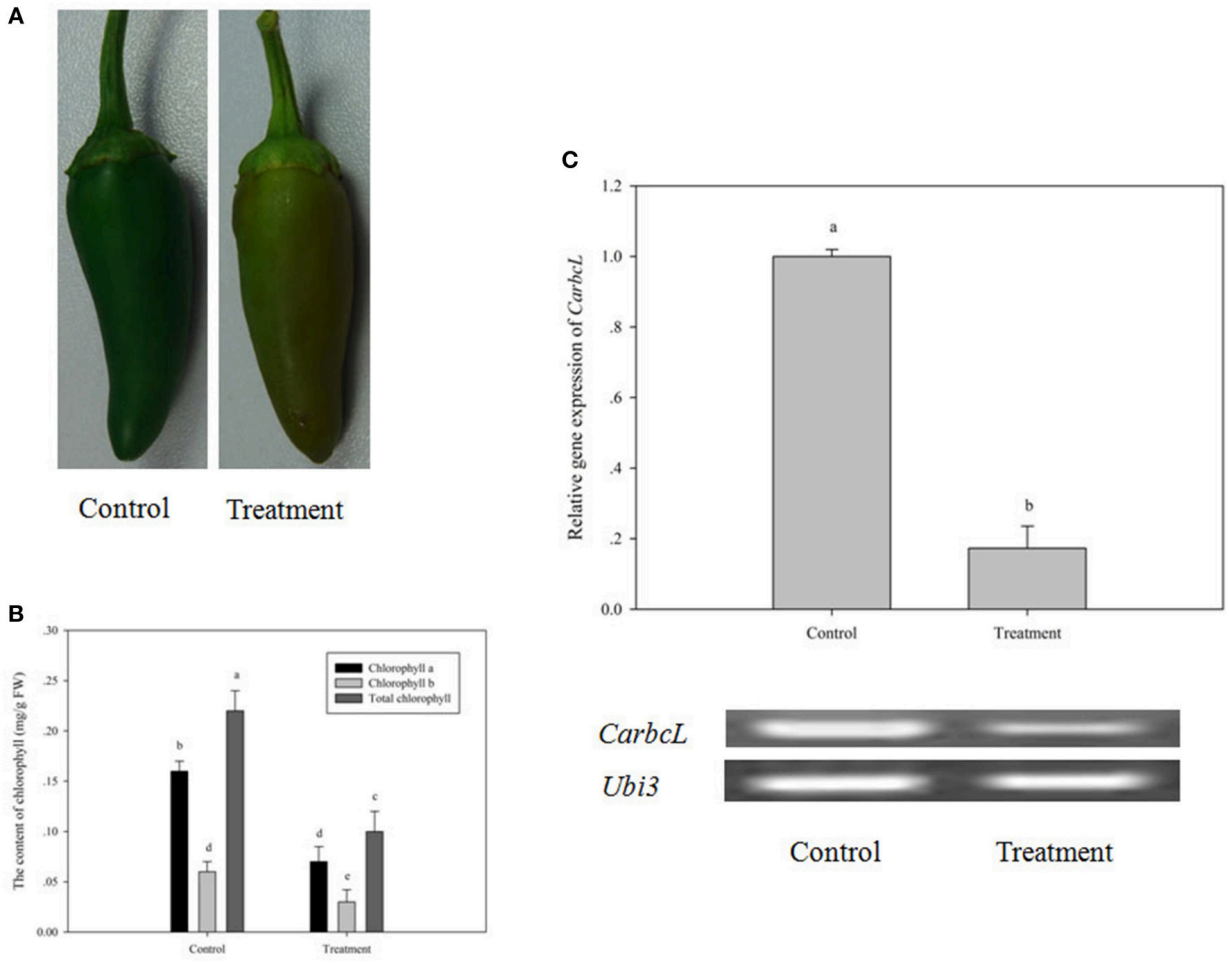
**Changes in fruit phenotype, chlorophyll content and ***CarbcL*** gene expression under low light**. **(A)** Phenotypic differences in fruits of the control and treatment groups. **(B)** Differences in the chlorophyll content of fruits in the control and treatment groups. **(C)** Gene expression differences in fruits of the control and treatment groups; Control: the control group (Fruits with no shade stress), Treatment: the treatment group (Fruits with shade stress). LSR analysis at 5% is shown in lowercase letters (a and b). Means with the same letter are not significantly different. All experiments were carried out in triplicate.

To further confirm the relationship of C*arbcL* gene and metabolic control of chlorophyll in pepper fruits, the initial experiment was repeated (a phenomenon of the green color of fruit fading under fruit shade stress). Specifically, pepper fruits were shaded by bagging for 7 days to renew shade stress for pepper fruits and to observe the change of pepper fruit color, the content of chlorophyll, and *CarbcL* mRNA expression level. As shown by the experimental results, after pepper fruits were shaded by bagging for 7 days, a greater and an obvious change had taken place in fruit color (Figure 6A). Comparing with the control, the color of fruit changed from dark green to a pale green (Figure 6A). Meanwhile, by determining the content of chlorophyll, it was found the content of chlorophyll decreased a lot (Figure 6B). Additionally, *CarbcL* gene expression level was significantly reduced in the treatment fruits (Figure 6C). Therefore, it was shade stress that the expression of *CarbcL* gene was repressed, thereby resulting in the degradation of chlorophyll to cause a phenomenon of fruit fading. This provides further evidence that *CarbcL* is involved in the metabolic control of chlorophyll and that *CarbcL* is a light-sensitive gene.

## Discussion

In our experiment, the *CarbcL* gene was cloned and characterized. Experimental results showed that the gene was related to chlorophyll in pepper fruits, and it is a RuBisCo large-subunit gene (*rbcL*). We know that RuBisCo is a plant metabolic enzyme that converts carbon dioxide from the atmosphere into organic carbon in the rate-limiting step of the Calvin cycle. It specifically catalyzes the carboxylation of d-ribulose-1,5-bisphosphate to form an unstable 6-carbon intermediate (Raven, 2013). In higher plants, RuBisCo is composed of eight small subunits encoded by a nuclear multigene family (*rbcS*) (Dean et al., 1989) and eight large subunits encoded by a single gene (*rbcL*) in the chloroplast genome (Rodermel et al., 1996). RuBisCo content increases rapidly during leaf expansion, reaches to maximum level at maturation, and then declines gradually during senescence stage (Imai et al., 2005; Suzuki et al., 2009b). A decrease in RuBisCo activity is a hallmark of senescence (Secor et al., 1984; Ford and Shibles, 1988; Crafts-Brandner, 1992). Light affect expression of *rbcL* gene stimulates *rbcL* transcription and increases the transcription of *rbcL* (Kim et al., 2002; Sun et al., 2014). Low light had significant effect on RuBisCo; it down-regulated the gene expression of RuBisCo at transcript levels and resulted in the loss and inactivation of RuBisCo large subunit (Sui et al., 2011; Sun et al., 2014).

By a series of experiments, the *CarbcL* gene was screened and cloned in pepper fruits by cDNA-AFLP under shade stress. Bioinformatics analysis indicated that *CarbcL* gene is associated with the metabolic control of chlorophyll. Silencing of *CarbcL* through VIGS technology in pepper fruits caused green fruits to fade early and turn red (Figure 4), and the expression level of *CarbcL* was strikingly reduced in the silenced lines (Figure 5A). Furthermore, the expression of *CarbcL* in shade-stressed fruits was very low (Figure 6C) during color changing from dark green to light green (Figure 6A) and the chlorophyll content considerably reduced (Figure 6B). The above experiment proved that *CarbcL* played an important role in the metabolic control of chlorophyll in pepper fruits. It was also found that capsanthin content was slightly reduced when *CarbcL* was silenced in pepper fruits (Figure 5C). This confirmed that inhibition of *CarbcL* gene caused not only chlorophyll degradation (Figure 5B) but also capsanthin content reduction (Figure 5C). Furthermore, *CarbcL* could not express normally under shade stress and shade stress caused chlorophyll degradation of fruits (Figure 6). These results further confirmed that *CarbcL* controlled chlorophyll metabolism in pepper fruits and is a light-sensitive gene. It was also noteworthy that *SGR* gene was not screened from all the differentially expressed fragments in our experiments. This showed that *SGR* gene did not play an important role in the metabolic control of chlorophyll under shade stress in pepper, although the *SGR* protein affects chlorophyll degradation during natural and dark-induced leaf senescence in rice (Jiang et al., 2007; Aubry et al., 2008; Barry, 2009). The results of current study will provide platform for future studies and have elucidated the regulatory mechanism that how low light (shade) influences fruit color during pepper fruit development.

## Conclusions

By cloning and characterization of the *CarbcL* gene, it was found that *CarbcL* participated in the metabolic control of chlorophyll. The gene (*CarbcL*) is very similar to Ribulose-1,5-bisphosphate carboxylase/oxygenase (*RuBisCo* large subunit). The function of the *CarbcL* gene was identified through virus-induced gene silencing (VIGS). The fruit color was changed completely from green to red except for some residue of green fleck when *CarbcL* gene was silenced, whereas the green color of fruits had not fully faded in the control group and the empty vector group. In the combine determination of chlorophyll content, *CarbcL* was found to be involved in the metabolic control of chlorophyll in pepper fruits. It was also found that the capsanthin content decreased appreciably after *CarbcL* gene silencing. It was further confirmed that *CarbcL* involved in the synthesis of chlorophyll and capsanthin. Under the shade stress, it was also found that *CarbcL* gene was repressed and caused chlorophyll degradation. This provides further evidence that *CarbcL* is involved in the metabolic control of chlorophyll and that *CarbcL* is a light-sensitive gene.

### Conflict of interest statement

The authors declare that the research was conducted in the absence of any commercial or financial relationships that could be construed as a potential conflict of interest.
