# Supplementary material for: Cloning and characterization of the CarbcL gene related to chlorophyll in pepper (Capsicum annuum L.) under fruit shade stress
Source: Front Plant Sci. 2015 Oct 13;6:850. doi: 10.3389/fpls.2015.00850 (PMC4602107; doi:10.3389/fpls.2015.00850)
Supplement: Supplementary file 1 [file Table1.DOCX]

Supplementary Table 1. Sequences of the primers used for selective amplification

| Primer name | Sequence (5’－3’) |
| --- | --- |
| SA01-F | gactgcgtaccaattcaac |
| SA02-F | gactgcgtaccaattcaag |
| SA03-F | gactgcgtaccaattcaca |
| SA04-F | gactgcgtaccaattcacc |
| SA05-F | gactgcgtaccaattcacg |
| SA06-F | gactgcgtaccaattcact |
| SA07-F | gactgcgtaccaattcagc |
| SA08-F | gactgcgtaccaattcagg |
| SA09-R | gatgagtcctgagtaacaa |
| SA10-R  SA11-R | gatgagtcctgagtaacac  gatgagtcctgagtaacag |
| SA12-R | gatgagtcctgagtaacat |
| SA13-R | gatgagtcctgagtaacta |
| SA14-R | gatgagtcctgagtaactc |
| SA15-R | gatgagtcctgagtaactg |
| SA16-R | gatgagtcctgagtaactt |

Primers (SA01-F, SA02-F, SA03-F, SA04-F, SA05-F, SA06-F, SA07-F and SA08-F) were combined with primers (SA09-R, SA10-R, SA11-R, SA12-R, SA13-R, SA14-R, SA15-R and SA16-R) respectively to form 64 primer combinations.
