# Supplementary material for: Cloning and characterization of the CarbcL gene related to chlorophyll in pepper (Capsicum annuum L.) under fruit shade stress
Source: Front Plant Sci. 2015 Oct 13;6:850. doi: 10.3389/fpls.2015.00850 (PMC4602107; doi:10.3389/fpls.2015.00850)
Supplement: Supplementary file 2 [file Table2.DOCX]

Supplementary Table 2. Homologies of cDNA-AFLP fragment expressed at shading of pepper fruit

| TDFs | Accession No. | Length/bp | Homology | Identify | Expression pattern |
| --- | --- | --- | --- | --- | --- |
| CaFS01 | KT779501 | 710 | Solanum tuberosum E3 ubiquitin-protein ligase | 94% | D |
| CaFS02 | KT779502 | 776 | Solanum lycopersicum alkaline/neutral invertase | 99% | D |
| CaFS03 | KT779503 | 485 | Solanum tripartitum (rbcL) gene | 99% | U |
| CaFS04 | KT779504 | 643 | Nicotiana tabacum heat shock protein 90 | 90% | U |
| CaFS05 | KT779505 | 656 | Coffea liberica 26S ribosomal gene | 99% | U |
| CaFS06 | KT779506 | 661 | Coffea liberica isolate CCC1025 26S ribosomal RNA gene | 99% | D |
| CaFS07 | KT779507 | 701 | Capsicum annuum stress-induced protein 15 mRNA | 93% | U |
| CaFS08 | KT779508 | 440 | Physalis heterophylla 18S ribosomal RNA gene | 99% | D |
| CaFS09 | KT779509 | 643 | Nicotiana tabacum heat shock protein 90 mRNA | 84% | D |
| CaFS10 | KT779510 | 625 | Solanum lycopersicum GRAS2 protein (GRAS2), mRNA | 92% | U |
| CaFS11 | KT779511 | 533 | Solanum lycopersicum DNA-binding protein, mRNA | 84% | D |
| CaFS12 | KT779512 | 357 | Solanum triflorum 26S ribosomal RNA gene | 100% | D |
| CaFS13 | KT779513 | 271 | Physalis heterophylla ribosomal RNA gene | 99% | U |
| CaFS14 | KT779514 | 471 | Solanum tuberosum ethylene-responsive transcription factor 3-like, mRNA | 81% | U |
| CaFS15 | KT779515 | 521 | Capsicum chinense auxin and ethylene responsive protein mRNA, complete cds | 99% | U |
| CaFS16 | KT779516 | 643 | Nicotiana tabacum heat shock protein 90 mRNA | 84% | U |
| CaFS17 | KT779517 | 651 | Solanum lycopersicum HTC in leaf mRNA | 91% | U |
| CaFS18 | KT779518 | 576 | Solanum tuberosum domain-containing protein mRNA | 97% | D |
| CaFS19 | KT779519 | 533 | Solanum tuberosum DNA-binding protein mRNA | 86% | U |
| CaFS20 | KT779520 | 364 | Solanum triflorum 18S ribosomal RNA gene | 99% | U |
| CaFS21 | KT779521 | 643 | Nicotiana tabacum hsp90 mRNA for heat shock protein 90 | 86% | D |
| CaFS22 | KT779522 | 576 | Lycopersicon esculentum anti-PCD protein mRNA | 91% | U |
| CaFS23 | KT779523 | 338 | Solanum lycopersicum HTC in fruit cDNA | 86% | U |
| CaFS24 | KT779524 | 338 | Solanum lycopersicum HTC in fruit cDNA | 86% | U |
| CaFS25 | KT779525 | 674 | Solanum triflorum 18S ribosomal RNA gene | 99% | U |
| CaFS26 | KT779526 | 804 | Populus trichocarpa hypothetical protein mRNA, | 86% | U |
| CaFS27 | KT779527 | 494 | Solanum tuberosum transcription elongation factor mRNA | 98% | U |

Note：CaFS01—CaFS27 were differently expressed bands under Capsicum fruit shading among 80 bands; D showed down-regulated expression；U showed up-regulated expression; CaFS03 was the band of the research result of Blastn.
